# Anti-Pathogenic Functions of Non-Digestible Oligosaccharides In Vitro

**DOI:** 10.3390/nu12061789

**Published:** 2020-06-16

**Authors:** Mostafa Asadpoor, Casper Peeters, Paul A. J. Henricks, Soheil Varasteh, Roland J. Pieters, Gert Folkerts, Saskia Braber

**Affiliations:** 1 Division of Pharmacology, Utrecht Institute for Pharmaceutical Sciences, Faculty of Science, Utrecht University, Universiteitsweg 99, 3584 CG Utrecht, The Netherlands; m.asadpoor@uu.nl (M.A.); Casper.peeters@live.nl (C.P.); P.A.J.Henricks@uu.nl (P.A.J.H.); soheil.varasteh@gmail.com (S.V.); g.folkerts@uu.nl (G.F.); 2 Division of Medicinal Chemistry and Chemical Biology, Utrecht Institute for Pharmaceutical Sciences, Faculty of Science, Utrecht University, Universiteitsweg 99, 3584 CG Utrecht, The Netherlands; R.J.Pieters@uu.nl

**Keywords:** non-digestible oligosaccharides, bacteria, bacterial growth, biofilm, adhesion, surface charge, chemical structure, HMOs, in vitro

## Abstract

Non-digestible oligosaccharides (NDOs), complex carbohydrates that resist hydrolysis by salivary and intestinal digestive enzymes, fulfill a diversity of important biological roles. A lot of NDOs are known for their prebiotic properties by stimulating beneficial bacteria in the intestinal microbiota. Human milk oligosaccharides (HMOs) represent the first prebiotics that humans encounter in life. Inspired by these HMO structures, chemically-produced NDO structures (e.g., galacto-oligosaccharides and chito-oligosaccharides) have been recognized as valuable food additives and exert promising health effects. Besides their apparent ability to stimulate beneficial microbial species, oligosaccharides have shown to be important inhibitors of the development of pathogenic infections. Depending on the type and structural characteristics, oligosaccharides can exert a number of anti-pathogenic effects. The most described effect is their ability to act as a decoy receptor, thereby inhibiting adhesion of pathogens. Other ways of pathogenic inhibition, such as interference with pathogenic cell membrane and biofilm integrity and DNA transcription, are less investigated, but could be equally impactful. In this review, a comprehensive overview of In vitro anti-pathogenic properties of different NDOs and associated pathways are discussed. A framework is created categorizing all anti-pathogenic effects and providing insight into structural necessities for an oligosaccharide to exert one of these effects.

## 1. Introduction

In recent years, there has been a growing interest in functional foods/nutraceuticals that have the ability to enhance human health, resulting in one of the leading trends in today’s food industry. Dietary carbohydrates, especially non-digestible oligosaccharides (NDOs), have been introduced as functional food ingredients. NDOs are known to selectively promote the growth and/or activity of beneficial bacteria in the gut, especially *Lactobacilli* and *Bifidobacteria* and, therefore, recognized as prebiotics [1]. To reach and be effective in the large intestine, NDOs are resistant to hydrolysis by intestinal digestive enzymes in the upper part of the intestines.

There is a great body of evidence that health-promoting effects of NDOs are not limited to shaping the intestinal microbiota and the microbiota-associated immune responses, but also include microbiota-independent effects on epithelial and immune cells [2]. It has been described that supplementation of NDOs to the diet in early life can decrease the development of diseases, such as allergies [3]. However, NDOs can also induce therapeutic effects in different inflammatory diseases later in life, including colitis, lung emphysema, cancer and HIV [4]. Recently, there has been particular scientific interest in the anti-pathogenic properties of NDOs for treatment (or prevention) of several kinds of infections, including gastrointestinal and respiratory infections [5,6]. Especially, antibiotic-resistant bacteria pose a great threat to human health and are associated with a major cause of morbidity and mortality worldwide [7]. Adhesion to host proteins (saccharides patterns) and biofilm development are thought to be two important pathogenic mechanisms. Bacterial biofilm formation is associated with a wide range of infections and reduces pathogenic susceptibility to antibiotic treatment. The multicellular nature of biofilms prevents the penetration of antimicrobial agents. Aggravation of antibiotic resistance among pathogenic species has urged development of alternative treatments for infections [8].

The microbiota protect against infections by promoting beneficial bacteria, such as *Bifidobacterium* and *Lactobacillus*, by inhibiting pathogenic bacteria or by orchestrating appropriate immune responses, therefore NDOs can play an important role in treating infections [9]. This topic has been extensively reviewed in recent publications [10,11,12].

NDOs can also act as anti-adhesives to selectively prevent adhesion of certain pathogen species to human cells and to mucin. For their adhesion-inhibiting properties, NDOs rely on structural similarity with oligosaccharide patterns presented on proteins on the host cell surface [13]. These patterns are essential for fimbria/pili-mediated pathogenic adhesion, allowing for anti-pathogenic capability termed receptor-mimicry [14,15,16]. In addition, it has been reported that NDOs possess anti-biofilm activity against different pathogenic microbes. NDOs can inhibit the development of pathogenic infection of the intestine before pathogen adhesion [14,17,18] or during one of the initial stages of biofilm formation [19,20,21] through direct interaction with pathogens.

Human milk contains a large amount of structurally diverse oligosaccharides, termed human milk oligosaccharides (HMOs), which represent the first prebiotics that humans encounter in life. Each structurally defined HMO might have a distinct functionality related to their anti-pathogenic properties. Inspired by the prebiotic and anti-pathogenic potential of HMOs, similar oligosaccharide structures were tested for their anti-pathogenic capability [14]. Some of the oligosaccharides produced are based on monosaccharides also present in HMOs, such as galactose (Gal) in galacto-oligosaccharides (GOS) and N-acetylglucosamine (GlcNAc) in chito-oligosaccharides (COS). Other commercial oligosaccharides with anti-pathogenic potential include mannan-oligosaccharides (MOS), alginate oligosaccharide (AOS), pectic oligosaccharides (POS) and fructo-oligosaccharides (FOS). These commercial NDOs can be obtained by direct extraction from natural sources or produced via enzymatic or chemical synthesis from saccharides [22]. There is a high structural diversity amongst these NDOs and depending on their key characteristics, such as monosaccharide components, charge, degree of polymerization (DP) and degree of acetylation (DA), these oligosaccharides elicit anti-pathogenic effects in a variety of ways.

In this review, an extensive overview of the anti-pathogenic effects of different NDOs and their postulated mechanisms are addressed. Herein, the focus lies on direct interaction of oligosaccharides with pathogens or components of the biofilm. Since the NDO-induced effects on the microbiota and microbiota-generated metabolites cannot be neglected in vivo, only In vitro studies are included. A framework is created categorizing all anti-pathogenic effects of relevant NDOs and providing insight into the structural requirements for an oligosaccharide to exert one of these effects.

## 2. Human Milk Oligosaccharides

### 2.1. Structure

**Figure 1:**
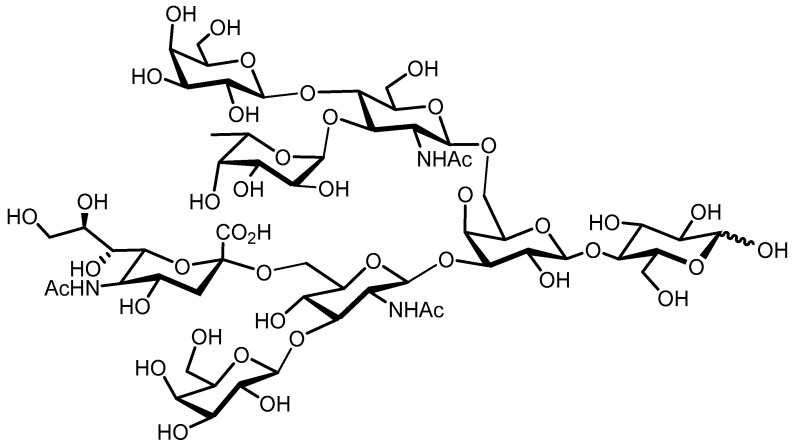
Exemplary structure of an HMO.

HMOs are soluble complex and diverse sugars containing Gal, Glc, fucose (Fuc), N–acetylglucosamine (GlcNAc), or sialic acid (Neu5Ac) monosaccharides. In the mammary glands, the HMOs are biosynthesized with the formation of a lactose core from Glc and Gal catalyzed by β–galactotransferase in the presence of α–lactalbumin. Galactose can be elongated enzymatically by β1–4 linkage to N-acetyllactosamine or by β1–3 linkage to lacto-N-biose. The core HMO structure can be further elongated by the addition of N-acetyllactosamine and lacto-n-biose units by β1–6 and β1–3 linkages; Fuc connected with α1–3, α1–2, or α1–4 linkages; and/or sialic acid residues attached by α2–6 or α2–3 linkages at the terminal positions (Figure 1) [23]. Human milk contains three major HMO types: neutral (Fucosylated) HMOs (e.g., 2-fucosyllactose (2-FL)), neutral N-containing HMOs (lacto-N-tetraose (LNT)) and acidic (sialylated) HMOs (e.g., 3-sialyllactose (3-SL)) [24].

### 2.2. Anti-Pathogenic Functionalities HMO Mixtures

HMOs have been shown to interact with pathogenic bacteria in a variety of ways (Table 1). Depending on their structural characteristics, HMOs may interact with adhesion factors on the pathogenic surface, or penetrate and interact with elements of the pathogenic biofilm, inhibiting microbial adhesion and biofilm growth [25,26]. When pathogens bind specific HMOs that resemble saccharide structures on the epithelial cell surface, their capacity to adhere to epithelial cells is inhibited.

#### 2.2.1. Anti-Adhesion HMO Mixtures

HMO structures binding to pathogenic fimbriae by resembling patterns on epithelial cell surface receptors are called decoy receptors [25]. The ability of HMOs to influence pathogenic adhesion is influenced by a number of variables, such as the percentage of fucosylated or acidic oligosaccharides in the mixture (the fucosylated and sialylated fraction, respectively), oligosaccharide weight or the type of pathogen.

##### Neutral HMO Fraction—Fucosylated and Non-Fucosylated

Neutral HMO fractions are known to inhibit adhesion of pathogens to epithelial cells. As a whole, the neutral fraction of HMOs inhibits an *Escherichia coli* strain, which is specifically P-fimbriated. i.e., galabiose (or galactose) specific, indicating a mechanism of receptor-mimicry [27]. After separation of a neutral oligosaccharide mixture into a high-and low-molecular weight fraction (HMWF and LMWF, respectively), the two fractions showed varying adhesion-inhibiting potential depending on the pathogen strain. The HMWF showed inhibition of *Vibrio cholerae* adhesion, whereas the LMWF inhibited *Salmonella fyris* [17], which contrasts the finding that the LMWF more potently inhibits *Vibrio cholerae* adhesion [28]. Larger HMOs appear to have an advantage over smaller HMOs in inhibiting the adhesion of pulmonary infectious strains, such as *Haemophilus influenzae,* however, the effect is also dependent on the composition of the HMO mixture [29]. One factor affecting pathogen adhesion to epithelial cells is the fucosylation status of the HMO mixture. Oligosaccharides are fucosylated by FUT2 (α1,2–fucose) or FUT3 (α1,4–fucose) [25]. In general, α1,2–fucose patterns on epithelial cell exterior have been shown to protect epithelial cells from pathogenic infection by facilitating colonization of a layer of probiotic microbes [30,31]. In addition, locally secreted glycan molecules may inhibit pathogenic colonization through a decoy-receptor mechanism, although this mechanism has not been fully elucidated [31,32].

In accordance with anti-colonization functionalities exerted by native fucosyl-containing elements, α1,2–fucosylated HMOs have also shown to exert anti-adhesion effects. A large part of the neutral oligosaccharide fraction is made up of fucosylated oligosaccharides, which is usually present at the reducing end of the oligosaccharide sequence [33]. Small fucosylated HMOs inhibit enteropathogenic *Escherichia coli* (EPEC) adhesion, when added to a HEP-2 monolayer along with EPEC [34], while fucosylated oligosaccharides show superior adhesion inhibition of *Neisseria meningitidis* to salivary agglutinin [35]. Although these results point in the direction of receptor mimicry functionalities of the neutral fraction, the anti-pathogenic effect cannot be tracked to a specific saccharide structure as the molecular diversity in these mixtures is very high [36]. Testing of isolated HMOs gives a better indication of the inhibitory capacities compared to testing of HMOs in mixture, this is discussed in the *Isolated HMO structures* section.

##### Acidic HMO Fraction

The acidic fraction of HMOs consists of sialylated oligosaccharides, which are negatively charged at homeostatic pH. Sialylated HMOs are produced by the action of sialyltransferase enzymes resulting in α2,3 and α2,6–sialylated oligosaccharides [25]. Due to the charge residing on the sialylated oligosaccharides, and their consequent interaction with oppositely charged elements on the epithelial cell exterior, their adhesion-inhibiting effect is less dependent on pathogen type compared to the neutral fraction [17]. Similar to neutral HMO fractions, the acidic HMO fraction shows inhibitory potential towards pathogenic species expressing specific fimbrial types, such as P and CFA fimbriae-expressing *Escherichia coli*. The lack of inhibition of HMO’s for P-fimbriated *Escherichia coli* is clarified by the lack of affinity of the P-fimbrial lectin for sialylated oligosaccharides instead of the Galα1,4 Gal (galabiose) termini on the cell surface, which are involved in recognition and adhesion of P-fimbriated pathogen species [27].

#### 2.2.2. Other Anti-Pathogenic Mechanisms of HMO Mixtures

Group B *Streptococcus* (GBS), often associated with post-natal infection and mortality, and its interaction with different HMOs has been of significant scientific interest in recent years. Pooled HMOs were shown to inhibit GBS growth and biofilm formation, provoking an alteration in biofilm structure. A suggested reason for the antibiofilm activity of these oligosaccharides is interference with nutrient cross-membrane transport by adhesion to the pathogen exterior [21].

Additionally, pooled HMOs potentiated the bactericidal function of a select number of ribosome-targeting antibiotics, clindamycin and erythromycin especially, against antibiotic resistant GBS and *Acinetobacter baumannii*, without the protection of a biofilm [37]. It was hypothesized that this is due to increased permeability of pathogens, a mechanism potentiated by polymyxins [38]. Antibiotics inhibiting cell wall synthesis are not potentiated, while treating any pathogenic strain in combination with pooled HMOs.

Finally, pathogenic cellular invasion can be affected by the presence of an HMO mixture. Pooled HMOs can inhibit *Escherichia coli* invasion of epithelial bladder cells by over 80%. Reportedly, HMOs aid in the preservation of paxillin [39], which is associated with the promotion of cohesion of the epithelial cell monolayer as a focal adhesion molecule [40]. The cell-protecting effect is further substantiated by complete inhibition of UPEC-induced upregulation of MAPK signalling [39], an important apoptotic cascade. Wider employment of this anti-pathogenic effect requires additional research with a higher variety of cell lines and experimental set ups.

### 2.3. Anti-Pathogenic Functionalities Isolated HMO Structures

The apparent antimicrobial functionalities of HMOs encouraged investigation of a number of isolated HMO structures. Even though the HMO mixture consists of over 100 distinct structures [45], the pathogenic interactions of only a relatively small number of individual HMO structures have been investigated (Table 2).

#### 2.3.1. Neutral Isolated HMO Structures

An example of an isolated HMO widely studied for its interactions with pathogens is 2-fucosyl-lactose (2-FL). 2-FL (α–l–Fuc– (1→2) –β–d–Gal– (1→4) –d–Glc or, α–l–fucopyranosyl– (1→2) –β–d–galacto–pyranosyl– (1→4) –d–glucopyranoside) is the most abundant fucosylated HMO in breast milk and has in multiple instances been linked with anti-adhesive properties. 2-FL mimics the H-2 epitope on epithelial cells. This glycosylic structure is important to pathogenic adhesion to epithelial cells [36]. Through this mechanism, 2-FL was shown to inhibit the adhesion of *Campylobacter jejuni* [46], *Pseudomonas aeruginosa*, EPEC, *Salmonella enterica* [47], but not the adhesion of UPEC, *Vibrio cholerae* and *Salmonella fyris* [17]. Even though specificity of certain pathogens for the H-2 epitope has been confirmed [48], the biochemical origin of pathogenic affinity for 2-FL was not further elucidated.

Although anti-adhesion functionalities of 2-FL have been well-described, there is scarce information about other types of anti-pathogenic effects of 2-FL. For interaction with pathogens in a biofilm structure, it is suggested that the neutral state of 2-FL limits it from entering the biofilm; attachment of a cationic element to 2-FL enables the molecule to enter extracellular polymeric substances (EPS) structures and exert antibiofilm activity [49].

3-Fucosyllactose (3-FL) is another trisaccharide observed in human milk, which is different in structure from 2-FL as its assimilation involves the enzymatic function of 3-fucosyltransferase (attachment of fucose to the reducing Glc end) instead of 2-fucosyltransferase [25]. Like 2-FL, interaction with pathogens has been documented for 3-FL in a number of instances [17], though the concentration of 3-FL (0.44 g/L) is lower in human milk samples compared to 2-FL (2.74 g/L) [50]. 3-FL inhibits adhesion to a number of pathogens, including UPEC, *Salmonella fyris* [17], EPEC, *Campylobacter jejuni, Salmonella enterica* and *Pseudomonas aeruginosa* [47]. Inhibition of adhesion of UPEC and *Salmonella fyris* by 3-FL, but not by 2-FL, indicates the importance of the location of fucosylation, apparently influencing the pathogenic receptor binding to the HMO structures [17]. However, the alternative placement of fucosylation does not alter the antibiofilm activity, as 3-FL also seems unable to penetrate into biofilm structures [42].

Compared to the 2 most investigated HMO structures, 2-FL and 3-FL, isolated HMO structures of larger size tend to exert more anti-pathogenic characteristics. LNFP I for example, a monofucosylated LN(n)T isomer which carries its Fuc in an α1–2 linkage at the terminal Gal and is the second most prevalent HMO (after 2-FL) [50], shows a high anti-pathogenic potential. LNFP I can significantly reduce pathogenic growth of GBS, while also exerting some antibiofilm action against GBS. In comparison with the other LNFP (LNFP II and LNFP III), LNFP I exerts the strongest antimicrobial potential. In addition, the anti-pathogenic properties of single HMOs were found to be strain-specific [42].

#### 2.3.2. Acidic Isolated HMO Structures

The isolated structures described thus far are neutral, which make up a large fraction of all oligosaccharides present in human milk [51]. Sialylation of oligosaccharides produces a negatively charged entity under neutral conditions. However, this does not seem to affect their ability to inhibit pathogenic adhesion; inhibition of UPEC and *Salmonella fyris* by 3-SL, a sialylated oligosaccharide structure, is comparable to inhibition by 3-FL [17]. 6-SL has been shown to be effective in inhibiting pneumocyte invasion *Pseudomonas aeruginosa* strains [52], while larger sialylated human milk oligosaccharides, such as LS-tetrasaccharide a (LSTa) exhibit a strong antimicrobial activity against GBS [44].

#### 2.3.3. Fucosylated Oligosaccharides (FO)

Fucose is present in human milk and the proportion of fucosylated HMOs in term breast milk was recently reported as 35–50% [23]. FO are constructed covalently joining of the l-fucose molecules to other monosaccharides via glycosidic linkages. l-Fucose is abundantly present in brown algae, like *Fucus, Laminaria, Sargassum*, and *Undaria* spp, as a major constituent of fucoidan [53]. There is evidence supporting the inhibitory effects of fucoidan on Helicobacter infections by adhesion inhibition to mucosal surfaces [54]. Another study included fractions of HMOs, containing about 5–20 different high-mass glycans with different degrees of fucosylation, in a neoglycolipid array [55] and demonstrated that high-mass HMOs with oligovalent fucose can exhibit stronger binding capacities towards blood group—active mucin-type O-glycans compared with monovalent fucose HMOs. Furthermore, HMO fractions with the strongest binding capacities contained hepta-to decasaccharides expressing branches with terminal Lewis-b antigen or blood group H1 [55,56]. It has been recently proved that the presence of fucose alone does not correlate to antimicrobial activity, while the location and degree of fucosylation does play a key role in HMO antimicrobial activity [42].

## 3. Alginate Oligosaccharides

### 3.1. Structure

**Figure 2:**
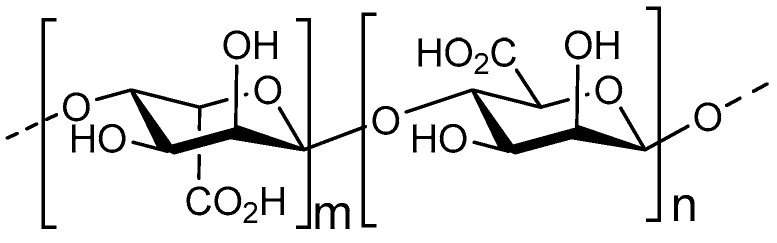
Structure of the main components of AOS; 1,4-linked β-d-mannuronic acid and 1,4-linked α-l-guluronic acid.

Alginate is a biopolymer, present in the cell walls of brown algae, and is composed of a sequence of two types of monosaccharides, 1,4–linked β–d–mannuronic acid (M) and 1,4 α–l–guluronic acid (G) (Figure 2) [57]. The M/G monosaccharide ratio, expressed as guluronic content (GC), is an important indication for antimicrobial functioning, as the G monomer has been shown to be preferred for cationic interaction as it is negatively charged [58]. From these polymers, AOS can be derived through enzymatic depolymerization or acid hydrolysis. So far, alginate biosynthesis has been detected in the *Azotobacter vinelandii* and the *Pseudomonas* species [59]. In *Pseudomonas aeruginosa* specifically, alginate biopolymers are an essential component of the biofilm EPS [60]. Alginates in these biofilms have slightly divergent structural characteristics, as they do not contain multiple G monosaccharides in sequence, termed G-blocks [61].

### 3.2. Anti-Pathogenic Functionalities

AOS have many antimicrobial functionalities (Table 3), and three main mechanisms of antimicrobial potential can be identified, all of which affect biofilm growth and development. First, AOS inhibit pathogenic swarming motility and proliferation. Second, AOS elicit a Ca^2+^ chelating effect in the presence of bio-alginates. Finally, AOS affect expression of quorum-sensing (QS) genes. Importantly, most of the anti-pathogenic effects of AOS have been elucidated studying its effect on *Pseudomonas aeruginosa*, due to the alginate presence in the *Pseudomonas aeruginosa* biofilm composition.

#### 3.2.1. Biofilm Inhibition

AOS have an extensive biofilm-inhibiting function. They inhibit pathogenic swarming and motility, important mediators in biofilm formation [62], in Gram-negative pathogenic strains *Pseudomonas aeruginosa*, *Escherichia coli* and *Proteus mirabilis* [63,64]. In *Pseudomonas aeruginosa*, inhibition of motility appears to be caused by AOS adhesion to the pathogenic exterior and flagella, along with a zeta-potential of the pathogenic cell surface by anionic AOS [65,66]. Inhibited motility and the resulting cellular aggregation has an inhibiting effect on the formation and growth of biofilms [67]. Even though alteration of surface-charge by AOS is limited to Gram-negative bacteria due to the polyanionic nature of the LPS layer of Gram-positive strains, AOS interaction with the LPS layer does induce biofilm-destructive bacterial aggregation of *Streptococcus mutans* [67]. Additionally, swarming of pathogenic cells and structured biofilm formation play an important role in the development of antibiotic resistance [68]. Indeed, AOS increase efficacy of several antibiotics against multidrug-resistant *Pseudomonas aeruginosa* [63,66,69]. Synergistic functionalities of AOS are not limited to antibiotics, as AOS adhesion to bacterial surface was also found to decrease colonization and biofilm formation in combination with an antibacterial and antifungal agent, triclosan [70].

#### 3.2.2. Metal ion Scavenging

AOS are potent Ca^2+^ scavengers. The Ca^2+^ scavenging activities of AOS inhibit biofilm formation in a number of ways. Ca^2+^ crosslinks alginate biopolymers, one of the major components of the EPS, improving structure and stability of the biofilm [71,72] and contributing to *Pseudomonas aeruginosa* resistance to antibiotics and elements of the immune system [73,74]. By scavenging alginate-associated Ca^2+^ in the biofilm, AOS remove these crosslinks and compromises EPS integrity and increases susceptibility of the biofilm to antibiotic treatment [63,66,69], which is in accordance with the observed higher affinity of Ca^2+^ for G-rich AOS [58]. Furthermore, considering Ca^2+^ availability induces alginate production *Pseudomonas aeruginosa* [75], AOS could also have a mediating function in the process of alginate-synthesis. The chelating properties are not universal to all bivalent cationic metals. Fe^2+^ for example, is another important factor in formation of the *Pseudomonas aeruginosa* biofilm and alginate production [76,77]. Contrary to Ca^2+^, Fe^2+^ is scavenged by neither AOS nor alginate [78].

#### 3.2.3. Quorum Sensing (QS) System Inhibition

Finally, AOS inhibit expression of QS genes in *Pseudomonas aeruginosa*. QS signalling is a cell-to-cell communication through extracellular exchange of signalling molecules to coordinate pathogenic behaviour [79]. The system is responsible for bacterial adaptation to the environment and plays a role in biofilm formation, swarming behaviour and antibiotic resistance of *Pseudomonas aeruginosa* [80]. Additionally, through QS signalling, biofilm structure and integrity is influenced, for example through production of eDNA [81], an important component of the EPS of *Pseudomonas aeruginosa*, acting as a cellular connector [82,83]. AOS inhibit production of two of the main components of the QS signalling system, acyl homoserine lactones (AHL) and C_4_–AHL and 3–oxo–C_12_–AHL [84]. This effectively inhibits pathogenic swarming motility and biofilm formation. As AOS do not show specific interactions with DNA [69], modulation of QS signalling molecule expression is believed to be achieved through interaction between AOS and C_4_–AHL and 3–oxo–C_12_–AHL. Consequently, the decline in intercellular signalling results in decreased synthesis of several virulence factors, such as elastase and pyocyanin [84]. Virulence factors exhibit important functionalities in biofilm persistence and antibiotic resistance [85]. Pyocyanin specifically inhibits production of eDNA [86]. Noticeably, AOS are able to make bacterial strains more susceptible to H_2_O_2_ by inhibiting QS-controlled virulence factors. QS affects the *Pseudomonas aeruginosa* resistance to H_2_O_2_ by production of antioxidants, such as superoxide dismutase and catalase, leading to the *Pseudomonas aeruginosa* resistance to the toxic free oxygen radicals [87,88].

## 4. Chito-Oligosaccharides/Chitosan Oligosaccharides

### 4.1. Structure

**Figure 3:**
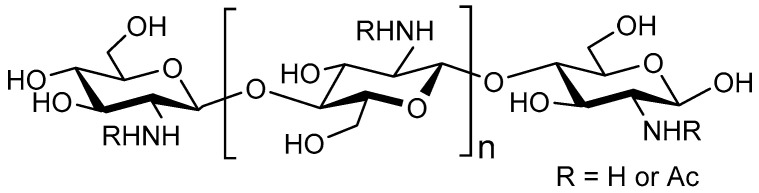
Structure of the main components of COS; with the monosaccharides N-acetylglucosamine (GlcN) and GlcNAc.

One of the most extensively investigated oligosaccharides are COS. COS are enzymatically or chemically processed products of chitin or chitosan polymers. Chitin is abundantly present in crustacean or arthropodic shells, while chitosan is more rare and must be extracted from cell walls of specific fungi [89]. Crustacean and arthropodic chitin consists of β–1,4–linked *N*-acetyl-d-glucosamine (GlcNAc), whereas chitosan consists of GlcNAc and the deacetylated form β–1,4–linked d–glucosamine (GlcN) (Figure 3). Chitin and chitosan copolymers are distinguished based on their DA: a DA of >70% usually refers to chitin, whereas a DA of <30% refers to chitosan [90]. The large chitin or chitosan polymers extracted from these biological sources, can be chemically or enzymatically hydrolysed into COS, with DP <20 and molecular weight of <3900 Da [91,92]. COS is highly soluble in a slightly acidic pH due to the charged state of the amine moiety (cationic nature) [93,94,95]. Chemically or enzymatically transformed COS is highly heterogenous with respect to DP and DA while the acetylation pattern (AP) can only be controlled to a certain extent [92,96]. Their enzymatic transformation allows for a limited control of the acetylation pattern [97]. GlcNAc is a ligand of F1C fimbriae in UPEC strains, involved in adhesion [98]. Nevertheless, antimicrobial effects of chitosan-based COS have been more extensively studied than chitin-based COS related to their increased solubility and cationic nature, making them more viable pharmacological prospects [90,99,100].

### 4.2. Anti-Pathogenic Functionalities

Ever since the antimicrobial activities of chitosan were first recognized [101], a wide range of studies have been conducted aiming to elucidate different antimicrobial pathways. A summary of the key antimicrobial activities of COS is presented in Table 4. First, COS have the potential to disrupt the bacterial cell membrane. Additionally, the surface-associating properties of COS can inhibit adhesion of pathogenic bacteria to host cells. COS also exhibit some antibiotic-potentiating properties and can inhibit RNA transcription in Gram-negative species. Importantly, different anti-pathogenic effects elicited by COS are often ambiguous amongst different sources. For example, the antimicrobial characteristics of COS are stronger against Gram-negative [95,102,103,104] or Gram-positive [93,94,105] bacterial strains, depending on the source.

#### 4.2.1. Cell Membrane Disruption

Polymeric molecules bearing a cationic charge are known to adhere to Gram-negative bacterial cell surfaces by ionic interactions with anionic lipopolysaccharide patterns [106]. Polyethylenimine, for example, is of great interest in pharmaceutical research for its polycationic functionalities and capability [107,108]. For cationic glucosamine components, present in COS, a similar mechanism is proposed, creating an impermeable cationic oligosaccharide layer around the bacteria [102,109] Concomitantly, chitosan adherence to bacterial cell surface promotes leakage of electrolytes and metal ions from the bacterial lumen [110,111,112]. Metal ions and other nutrients essential for bacterial proliferation are unable to diffuse across the bacterial membranes [102,112]. Prolonged exposure to COS and the resulting osmotic imbalance results in inhibition of growth, cell swelling and, ultimately cell lysis [112,113]. The bactericidal activity of COS increases with an increased glucosamine share, and is greater than that of polymeric chitosan [114].

#### 4.2.2. Adhesion Inhibition

Unlike other types of oligosaccharides, the anti-adhesive properties of COS have been relatively poorly studied. Yet, COS (DP 4 >12, DA 15–65%) was found to be a potent, pathogen-specific inhibitor of EPEC adhesion, but not verotoxin-producing *Escherichia coli* (VTEC) [115,116]. Interestingly, although EPEC expressing F1C fimbriae were demonstrated to show affinity for GlcNAc, the DA hardly influences inhibition of pathogenic adherence [115]. An explanation for this is the abundance of strong ionic interactions mediated by the cationic amine moiety of glucosamine in addition to the GlcNAc recognition.

#### 4.2.3. Association with Bacterial DNA

Chitosan has a strong interaction with fungal and plant DNA, mediated by electrostatic interactions, resulting in inhibited mRNA transcription [117,118,119,120]. Chitosan only penetrates into bacterial cells after disruption/lysing of the bacterial membrane [112,113]. COS, on the other hand, is linked to binding to bacterial DNA independent of bacterial lysis, inhibiting DNA transcription. This effect is most potent with COS M_w_ ≤ 5000 [121]. At this size, COS is small enough to penetrate the bacterial membrane [94,122]. COS interference with DNA transcription is linked to a decreased alginate production and, thus, biofilm formation in *Pseudomonas aeruginosa* [88]. mRNA transcription in Gram-negative species, however, is not affected in a similar manner, as their thick peptidoglycan layer prevents cellular penetration of COS [94,104].

#### 4.2.4. Synergy with Antibiotic Treatment

Multi-drug resistance (MDR) is often achieved by bacteria by upregulation transmembrane multidrug efflux pumps [123]. COS sensitize multi-drug resistant *Staphylococcus aureus* and *Pseudomonas aeruginosa* to a number of common antibiotic formulations [88,124,125], possibly through formation of the aforementioned cationic oligosaccharide layer around bacteria, or ionic interactions with multidrug efflux pumps.

## 5. Fructo-Oligosaccharides

### 5.1. Structure

**Figure 4:**
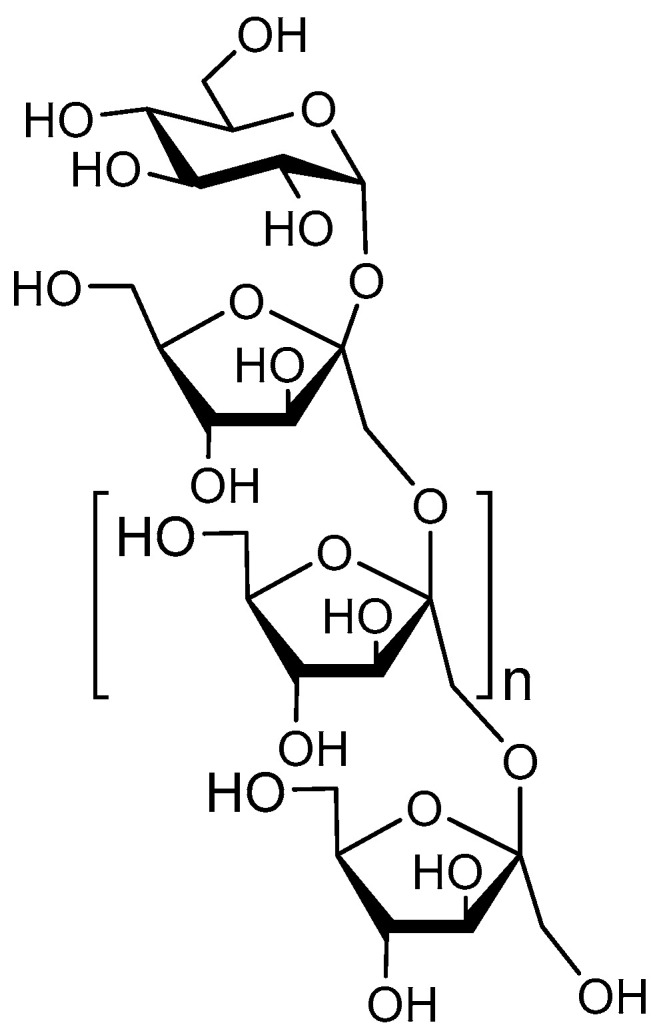
Structure of the main components of FOS; a glucose monomer, followed by an *n* number of fructose monomers in sequence.

FOS are a common component of a healthy diet [129] and are widely investigated for their prebiotic functionalities. Sucrose (a glucose-fructose disaccharide) is transformed into fructose by a transfructosylating enzyme. The FOS structure is characterized by a single sucrose monomer followed by a variable number of fructose monomers, sometimes in a 2→6 but often with a 2→1 linkage (Figure 4). FOS structures larger than DP10 are termed inulin.

### 5.2. Anti-Pathogenic Functionalities

Although FOS are renowned for their indirect anti-pathogenic functionalities, namely their prebiotic capacity, direct anti-pathogenic functionalities of FOS are not widely investigated. FOS and inulin were associated with pathogenic anti-adhesion. Inulin inhibited the adhesion of *Escherichia coli* to human epithelial cells and FOS decreased the ability of *Escherichia coli* Nissle 1917 to adhere to human intestinal epithelial cells [130,131]. In addition, FOS decreased the growth, biofilm formation, and motility behaviour of *Pseudomonas aeruginosa* PAO1, while inulin showed the opposite effects. The FOS-induced decrease in exotoxin A, a *P. aeruginosa* virulence factor, could be a possible mechanism for the reduction in pathogenicity [132].

A number of causes may potentially underlie the scarcity of the anti-pathogenic functionalities of FOS. As we have seen, an oligosaccharide can serve as a substrate for a bacterial adhesin involved in pathogenic adhesion and offers a predictive value for the anti-adhesive functionalities. So far, FOS has not been associated with targeting any specific pathogenic adhesins and the existence has only been theorized [131]. Secondly, unlike several other oligosaccharides, FOS lack functional groups capable of bearing a charge. For this reason, FOS do not engage in ionic interaction the way (partially) charged oligosaccharides.

## 6. Galacto-Oligosaccharides

### 6.1. Structure

**Figure 5:**
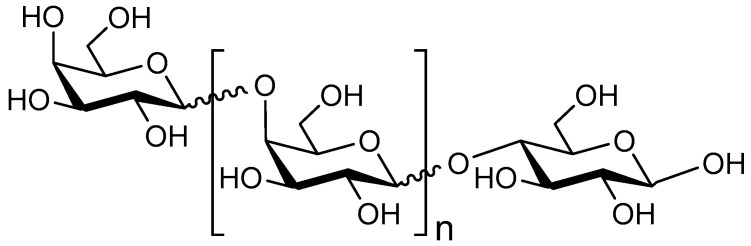
Structure of the main components of GOS; 1,4–linked and 1,6–linked β–galactose and a reducing-end glucose.

Although galactose is an important monosaccharide component of some HMOs, GOS are not a component of HMOs [25], but are known to mimic the biological effects of HMOs [133]. Commercially available GOS are most commonly composed of β–galactooligosaccharides instead of α–galactooligosaccharides and usually have a DP ranging from 2 to 6 [134]. Typically, commercial GOS mixtures are structurally heterogenous due to enzyme activity, as they feature different types of linkages between monosaccharides. Most of these linkages are of 1→4 (Figure 5) or 1→6 in nature. Often, the enzymes used produce different types of linkages within one oligosaccharide, resulting in a range of different oligosaccharides in a mixture [135]. Unfortunately, many experiments performed with GOS have no clear characterization of the linkages of the used oligosaccharide mixture, and often an indication of the suspected GOS linkages are provided as a suggestion. Additionally, other types of oligosaccharides can be galactosylated, adding galactose characteristics.

### 6.2. Anti-Pathogenic Functionalities

Two mechanisms of antimicrobial activity by GOS have been identified. GOS elicit anti-adhesive properties but can also inhibit host cell interaction with pathogenic toxins (Table 5).

#### 6.2.1. Adhesion Inhibition

GOS interactions are attributed to their association to specific pathogenic adhesins. Some parasites, such as *Entamoeba histolytica*, use β–galactose patterns on intestinal epithelial cells for lectin mediated adhesion [136,137]. For this reason, GOS have been subject to investigation for the identification of a similar effect [138,139]. GOS were first reported to reduce cellular adhesion of EPEC [138], and later the anti-adhesive effect of GOS was shown for a number of other pathogenic strains, such as *Salmonella typhimurium* [139]. The anti-adhesive effect of GOS on *Citrobacter rodentium* is dependent on adhesin expression, as deduced from a diminished antiadhesive effect of GOS after expression alteration of fimbria-mediated genes [140]. This further suggests an adhesin-specific anti-adhesive effect of GOS. However, GOS did not show an anti-adhesive and anti-growth effect against *Listeria monocytogenes* [141]. Until now, a specific interaction between GOS and a pathogenic adhesin has not been identified. Interestingly, GOS significantly inhibit cellular adhesion of *Cronobacter sakazakii* [142], a strain suggested to exert a fimbria-independent mechanism of cellular adhesion [143]. Therefore, the anti-adhesive activity of GOS could (at least in part) be fimbria-independent.

#### 6.2.2. Anti-Toxin Binding

Cholera toxin (Ctx) is produced and excreted by the *Vibrio cholerae* strain and binds to host cell surface GM-1 receptors, causing cellular salt and H_2_O excretion, resulting in diarrhea [144,145]. GM-1 receptors are lipid-conjugated oligosaccharides and contain a terminal galactose [146]. The GM-1 receptor, expressed on the membrane of intestinal epithelial cells, is responsible for Ctx entry into the host cell [147], although fucose binding is now also considered to be part of this [148]. GOS is hypothesized to bind to Ctx, inhibiting its GM-1 host cell entry mechanism [149] similar to dendritic GM1-oligoscaccharide compounds [150].

## 7. Mannan-Oligosaccharides

### 7.1. Structure

**Figure 6:**
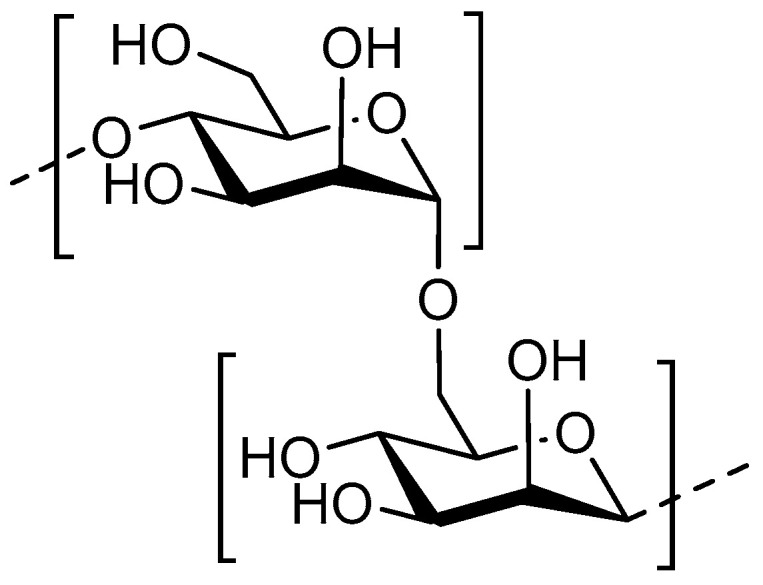
Structure of the main components of MOS; 1→4 linked d–mannose (**top**) and 1→6 linked D–mannose (**bottom**).

There are multiple ways of producing mannan-oligosaccharides (MOS). Previously, yeast products were harvested and directly applied for In vitro experimentation [151,152]. Nowadays, the most common ways of MOS production are chemical synthesis or autolysis of biopolymers extracted from yeast. Synthetically produced MOS can be structurally defined and nature of intergycosidic linkages can be determined [153]. Alternatively, isolation of autolysed yeast cell wall yields a heterogenous mixture of (branched) MOS, including 1→2, 1→4 and 1→6 d–mannose linkages [154]. The structure of MOS is shown in Figure 6. A drawback of MOS for pharmacological purposes is its branched nature and the unpredictability of the product structure after enzymatic production.

### 7.2. Anti-Pathogenic Functionalities

The mannose monosaccharides and MOS are well-known for their anti-adhesion capacity against pathogen adhesion, as summarized in Table 6.

#### Adhesion Inhibition

The mannose monosaccharide is an established and widely studied ligand for the FimH domain of type I fimbriae. The FimH domain of the type I fimbria is responsible for recognition of mannose patterns on host cell exterior and subsequent mannose-dependent pathogenic adhesion [155,156]. Type I fimbriae are commonly found in *Salmonella* spp. and *Escherichia coli* and play an important role in adhesion by binding to mannose patterns in host cell epithelial receptors [151,152,157]. It was previously shown that glycosides of mannose exhibit amplified anti-adhesive properties towards *Escherichia coli* compared to mannose monosaccharides, indicating the importance of a hydrophobic region in the vicinity of the mannose binding area for type I fimbria adhesion [158,159]. Mannose can bind to different FimH variants from different *Escherichia coli* pathotypes, concluding that mannose affinity for the FimH domain is independent of pathotype [160]. The mannose binding pocket of the FimH was later determined to be identical within different pathogenic species, including *Escherichia coli* and *Klebsiella pneumonia* [161]. As mannose glycosides have a significantly higher affinity for the *Klebsiella pneumonia*, it is likely that FimH structure varies between species and are also presented differently [161]. This difference is also reflected in superior *Escherichia coli* specificity for mono-or trimannose moieties [162,163]. Furthermore, significant reduction in the adherence of *Campylobacter jejuni* and *coli* to human epithelial cells was observed in the presence of MOS [163]. An overview of anti-adhesion activities is shown in Table 6. MOS binds to the FimH domain in competition with mannose patterns on host epithelial cells. This inhibits pathogenic adhesion by exerting a receptor-mimicking function [164,165]. Contrary to the mannose monosaccharide described earlier, addition of hydrophobic triethylene glycol to MOS (DP ≥ 3) does not increase the affinity for FimH compared to unconjugated MOS [160]. Inhibition of pathogenic adhesion by MOS is non-superior to inhibition by yeast cell wall, containing mannose biopolymers [166].

## 8. Pectic Oligosaccharides

### 8.1. Structure

**Figure 7:**
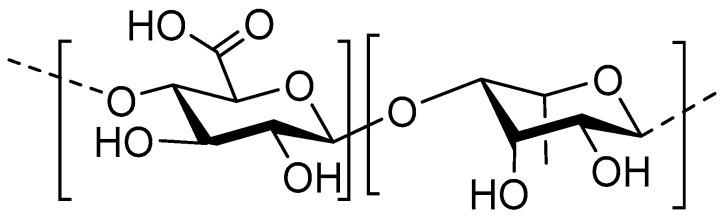
Structure of the main components of POS; a non-methylated d–galacturonic acid monomer (**left**) linked in a β1→4 fashion with a rhamnose monomer (**right**).

Pectin is a plant biopolymer, acting as a stabilizer for the cellulose network [169]. Pectin is a complex biopolymer made up of combined monosaccharides, most importantly (1→4) linked d–galacturonic acid (GalA). GalA is the main component of the pectin backbone, with a L-rhamnose content of 2–4% (Figure 7) [170]. GalA monosaccharides of pectin biopolymers are 6–methyl esterified to a certain extent, depending on the presence of pectin esterase in the source [171]. Much like acetylation of chitosan, the extent to which pectin is methylated dictates its function; low methylation ensures higher hydrophilicity and more interaction with cationic metal agents [172]. Depolymerization of pectin yields POS, which can due to the high diversity of the source polysaccharide, assume a high variety of forms. POS investigated for antimicrobial purposes are often depolymerized by enzymatic hydrolysis and are often derived from orange/bergamot peel [173,174]. Pectin found in bergamot peel is especially useful due to presence of ‘hairy’ and ‘smooth’ regions of GalA backbone. Hairy regions of pectin are equipped with arabinose, galactose, glucose, mannose and xylose elements [175]. This way, a large number of structurally different oligosaccharides can be synthesized from a single source.

### 8.2. Anti-Pathogenic Functionalities

POS have a wide range of antimicrobial activity as summarized in Table 7. POS have the capability to inhibit growth of several pathogens, inhibit adhesion of pathogenic bacteria and can also interact with pathogen-produced Shiga-like toxins (Stx).

#### 8.2.1. Growth Inhibition

Although multiple types of POS have shown to inhibit pathogenic growth, no mechanism has been determined thus far. Citric POS inhibit growth of a number of pathogenic strains, with superior efficacy of growth inhibition of low M_w_ POS for all inhibited strains [176]. The mechanism through which inhibition is achieved seems to be strain-dependent, as Gram-negative *Campylobacter jejuni* growth remains unaffected [177]. POS extracted from haw fruit show inhibition of pathogen growth at relatively low concentrations compared to citrus-extracted POS, although the mechanism through which inhibition is achieved remains unclear [178]. The induced strain-dependent growth inhibition could be related to increased radical scavenging abilities of charged POS. However, unlike COS and AOS, unspecific ionic interaction of charged carboxylic acid groups with the pathogenic exterior is unlikely to be the main source of pathogenic growth inhibition by POS, as it is strain-dependent.

#### 8.2.2. CO_2_ Radical Production

POS have shown to efficiently scavenge free radicals [179,180]. Free radicals, such as HO•, have a number of pathological effects, such as DNA damage and carcinogenesis [181,182]. Radical-scavenging substances have been widely studied for their attractive pharmacological properties [183]. Interestingly, HO• radical scavenging appears to trigger CO_2_•^−^ radical production by several different types of POS, which is hypothesized to inhibit *Staphylococcus aureus* and, less significantly, *Escherichia coli* growth [184]. However, due to the versatility of POS characteristics, the anti-pathogenic effect of CO_2_•^−^, if any, has not been successfully gauged.

#### 8.2.3. Adhesion Inhibition

POS inhibit adhesion of several kinds of pathogenic strains, most notably, *Escherichia coli*. The anti-adhesive mechanism referred to is inhibition of P-fimbria-mediated adhesion [185,186,187]. The extent of inhibition exhibited by POS seems to be beneficially influenced by high GalA content and low degree of 6-methyl esterification [188]. However, the P-fimbriae-specific inhibition of adhesion is not supported by the presence of a GalA binding pocket on these P-fimbriae [186,188]. For this reason, the exact mechanism of fimbria-specific inhibition of adhesion is still unclear. Additionally, high uronic acid content in POS and, consequently, higher ionic interactions between oligosaccharides and pathogens have been proposed to contribute to the anti-adhesion functionalities against Gram-positive *Staphylococcus aureus* bacteria. Interestingly, uronic acid-rich oligosaccharides did not prove effective for inhibition of adhesion of *Escherichia coli* [189].

#### 8.2.4. Inhibition of Toxin-Binding

Shigatoxigenic *Escherichia coli* produce Stx type 1 as well as type 2. The pentamer subunit of Stx, termed StxB interacts with a number host cell surface constituents, the main one globotriaosylceramide (Gb3), for epithelial cell internalization [190,191]. The Gb3 receptor is a lipid-conjugated oligosaccharide structure consisting of a Galα1,4Galβ1,4Glc trisaccharide [192]. Even though Stx type 1 and type 2 do not necessarily always use the same pathways to enter cells [193], POS inhibit the host cell uptake of both types of Shiga toxin in two ways. First, GalA-rich POS is associated with competitive binding of Gb3 with Shiga toxin. GalA inhibitory capacity of Gb3 is similar to that of its primary substrate [188]. Additionally, POS directly binds to Stx, due to structural similarities between POS and the galabiose receptor. This interaction inhibits Stx association to the Gb3 receptor, to a comparable extent to inhibition by the minimum receptor analogue galabiose (Galα1,4 Gal) [194], reducing host cell uptake of Stx [185]. Similarly, the Stx-binding capabilities of POS have been proposed to assist in inhibiting *Campylobacter jejuni* infiltration into host epithelial cells [177]. This interaction provides a long-term disabling effect and possibly even structural alteration of the bound toxin [188] which could prove useful in clinical application considering Stx, in conjunction to their ability to infiltrate host epithelial cells, also infiltrates underlying tissues [195].

## 9. Conclusions and Future Perspectives

**Figure 8:**
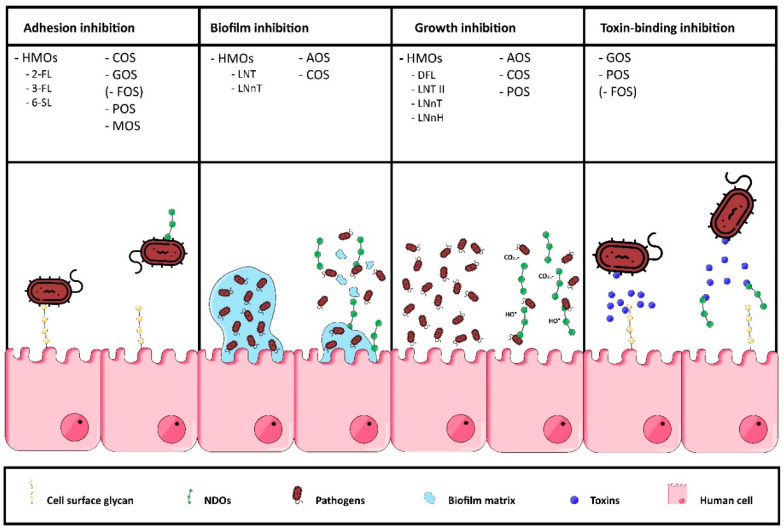
Schematic overview of the anti-pathogenic functionalities of NDOs in vitro. The first column shows that different NDOs (different HMOs, COS, GOS, FOS, POS and MOS) can serve as decoy receptors that competitively bind pathogens, which prevents pathogen adhesion to cell surface glycans. The second column indicates that several HMOs, AOS and COS can inhibit biofilm formation by penetrating and interacting with elements of the pathogenic biofilm. Multiple NDOs, such as, different HMOs, AOS, COS and POS, have shown to inhibit pathogenic growth, for example, by disrupting the bacterial cell membrane and/or by scavenging free radicals, such as HO•, which have a number of pathological effects (third column). The fourth column indicates that several NDOs (GOS, POS, FOS) can inhibit host cell interaction with pathogenic toxins.

In vitro investigation of NDOs has unveiled a wide range of anti-pathogenic functionalities, including anti-adhesion properties against pathogens, inhibition of biofilm formation, inhibition of specific pathogen growth and toxin-binding properties. An overview of these anti-pathogenic functionalities with corresponding NDOs is illustrated in Figure 8.

Most of the anti-pathogenic functionalities elicited by a specific oligosaccharide can be predicted by investigation of a number of characteristics, for example, the presence of a pathogenic adhesin or toxin that may bind to the carbohydrate sequence and/or potential charge of the oligosaccharide. However, structural features of oligosaccharides responsible for their adhesin-specific anti-adhesion properties are not necessarily related to mechanisms of other anti-pathogenic activities. Ionic interaction between charged oligosaccharides and pathogenic exterior can cause decreased motility and transport of nutrition, while some NDOs may electrostatically interact with intracellular DNA, inhibiting DNA-transcription.

Although several mechanisms of anti-pathogenic functioning have been identified, not all studies propose a clear explanation for the observed anti-microbial properties, and there are also contradictory reports concerning the antimicrobial potential of several NDOs against different types of microbial strains. A better characterization of the oligosaccharides in terms of DP, DA and monosaccharide sequence and testing a wider range of pathogens could assist in further uncovering details of anti-pathogenic functionalities. Glycan (or carbohydrate) arrays (e.g., using glycan probes) could also contribute to fast and high-throughput screening of protein-carbohydrate interactions with small amounts of carbohydrate ligands [196,197].

For clinical application, monotherapy with single adhesin-specific NDOs will have limited chance of successfully inhibiting pathogen adhesion, due to the variability of pathogenic adhesin expression. Rather, a mixture of NDOs with affinity for different adhesins could have a higher clinical applicability and in general, NDOs are not pure products, but are mixtures containing oligosaccharides of different DP.

For direct inhibition of pathogens, charged NDOs are more interesting to the clinical environment compared to uncharged NDOs due to lower pathogen-specificity, not relying on expression of a specific pathogenic adhesin. Non-food application, including local application via lung or skin, can be proposed for future investigation. Before we can make any statements about future applications requiring systemic delivery of NDOs, assessment of systemic stability, toxicity and immunogenicity of NDOs is needed.

In conclusion, versatility of antimicrobial effects, their unique ability to penetrate and inhibit biofilm structures and their limited side effects plea for oligosaccharides as a useful tool in the battle against emerging infections and antibiotic resistance. In addition, the effects of NDOs on promoting beneficial bacteria in the gut should not be neglected, since a well-balanced microbiota contributes to protection against infections by inhibiting pathogenic bacteria or by orchestrating appropriate immune responses.

## Figures and Tables

**Table 1:** Overview of the anti-pathogenic functionalities of HMO mixtures.

HMO Characteristics (Source)	[HMO]	Strains Used	Observed Effects	References
Breast milk collected from first and fourth week of lactation	1:2 dilution	***Gram-positive:*** *Streptococcus pneumoniae* ***Gram-negative:*** *Haemophilus influenzae*	Anti-adhesive effects against *Haemophilus influenzae* (HMWF) and *Streptococcus pneumoniae* (all HMOs)	[23]
Breast milk from healthy women collected 30 days after delivery	6 mg/mL	***Gram-negative:*** UPEC	Anti-adhesive effects of neutral fractions (high > low Mw)	[28]
Colostrum (d1–4), transitional (d12–17) and mature (d28–32) breast milk from healthy women	25–1200 μg/well (50 μL)	***Gram-negative:*** ETEC (CFA/I, CFA/II fimbriae), UPEC (P, P-like fimbriae)	Inhibition of hemagglutination by desialylated fraction associated with binding to P-fimbriae	[27]
Pooled transitional breast milk samples	20 g/L	***Gram-negative:*** *Neisseria meningitidis*	Inhibition of binding to pili by acidic HMO fraction	[35]
HMO fractions and modified HMO fractions from pooled human milk	1–2 g/L	Isolated, immobilized P-selectin	Interference acidic HMO fraction and P-selectin. Neutral HMOs show no interference.	[41]
Colostrum (different fractions) collected four days after delivery	1, 5, and 10 mg/mL	***Gram-negative:*** EPEC, *Vibrio cholerae*, *Salmonella fyris*	Anti-adhesive effects against *Salmonella fyris* (acidic, neutral, LMw), and *Vibrio cholerae* (neutral, hMw)	[17]
HMOs isolated from pooled human milk	15 mg/mL	***Gram-negative:*** UPEC	Inhibition of bacterial invasion but no anti-adhesive effects, protection	[39]
Breast milk from healthy women collected between 3 days and 3 months postnatal	5 mg/mL	***Gram-positive:*** GBS	Up to 40% growth inhibition	[21]
Breast milk from healthy women collected between 3 days and 3 months postnatal	5 mg/mL	***Gram-positive:*** GBS (CNCTC, GB590, GB2)	8–32× MIC reduction with antibiotics in combination with HMOs	[42]
Breast milk from healthy women collected between 3 days and 3 months postnatal	5 mg/mL	***Gram-positive:*** GBS, *Staphylococcus aureus* ***Gram-negative:*** *Acinetobacter baumannii*	GBS and *Staphylococcus aureus* biofilm inhibition, no antimicrobial effect	[43]
Breast milk from healthy women collected between 3 days and 3 months postnatal	5 mg/mL	***Gram-negative:*** GBS (GB590, GB2)	HMO mixture more effective inhibition of pathogen growth and viability reduction than isolated oligosaccharides	[44]

**Table 2:** Overview of the anti-pathogenic functionalities of isolated HMOs.

HMO Characteristics	[HMO]	Strains Used	Observed Effects	References
2-FL, 3-FL, 3-SL, 6-SL	2-FL = 2.5 mg/mL 3-FL = 0.5 mg/mL 3′-SL = 0.1 mg/mL 6′-SL = 0.3 mg/mL	EPEC, *Vibrio cholerae*, *Salmonella fyris*	Anti-adhesive effect of 6-SL and 3-FL against *Escherichia coli* and *Salmonella fyris*	[17]
Synthesized 2-FL, 3-FL	10 mg/mL	***Gram-negative:*** *Campylobacter jejuni*, EPEC, *Salmonella enterica, Pseudomonas aeruginosa*	Differential anti-adhesive effect	[47]
3-SL and 6-SL	2 μg/mL–1 mg/mL	***Gram-negative:*** *Pseudomonas aeruginosa*	Dose-dependent inhibition by 6-SL of pneumocyte invasion (lung)	[52]
3-SL, 6-SL, LNT, LSTa, LSTc, DSLNT	5 mg/mL	***Gram-positive:*** GBS	Inhibition of biofilm production and growth by larger sialylated oligosaccharides	[44]
2-FL, 3-FL, DFL, LNT, LNnT, LNFP I, LNFP II, LNFP III, LNT II, para-LNnH, LNnH	5 mg/mL	***Gram-positive:*** GBS	Strain-specific antimicrobial activity, no biofilm inhibition, fucose not involved in antimicrobial function	[42]
2-FL1-N-2-FL	-	***Gram-positive:*** GBS	No antimicrobial or antibiofilm activity of 2-FL Antimicrobial/antibiofilm activity due to cationic moiety of 1-N-2-FL	[49]

**Table 3:** Overview of the anti-pathogenic functionalities of AOS.

AOS Characteristics	[AOS]	Strains Used	Observed Effects	References
***GC:*** 90–95% (OligoG CF 5/20), 46%, 0% ***M_W_:*** 2.6 kDa	2%, 6%, 10%	**Gram-negative:** *Pseudomonas aeruginosa, Proteus mirabilis, Escherichia coli*	Inhibition of motility and biofilm formation, antibiotic synergy	[63]
OligoG CF-5/20	2%, 6%, 10%	**Gram-negative:** *Pseudomonas aeruginosa, Acinetobacter* *baumannii*	Structural interference biofilm formation, antibiotic synergy	[64]
OligoG CF-5/20	2%, 6%, 10%	**Gram-negative:** *Porphyromonas gingivalis* **Gram-positive:** *Streptococcus mutans*	Triclosan synergy	[70]
OligoG CF-5/20	0.2%–10%	***Gram-negative:****Pseudomonas aeruginosa, Burkholderia* spp.	Inhibition of pathogenic cell motility	[65]
Alginate-derived oligosaccharides M/G: 2.28 ***M_W_:*** 300 kDa	2 mg/mL	**Gram-negative:** *Pseudomonas aeruginosa*	Antibiotic synergy, anti-biofilm effect, decrease in virulence, increase in susceptibility to H_2_O_2_ of pathogen	[88]
OligoG CF-5/20	5–100 mg/mL	**Gram-negative:** *Pseudomonas aeruginosa* **Gram-positive:** *Streptococcus mutans*	Cellular aggregation of *S. mutans* and *P. aeruginosa* and binding of OligoG CF-5/20 to *P. aeruginosa.* Anti-microbial effects are not related to structural alterations in LPS or cell permeability	[67]
OligoG CF-5/20	2%	**Gram-negative:** *Pseudomonas aeruginosa*	Antibiotic synergy	[66]
OligoG CF-5/20	-	**Gram-negative:** *Pseudomonas aeruginosa*	Inhibition of QS-signaling	[84]
OligoG CF-5/20	0.5%, 2%, 6%	**Gram-negative:** *Pseudomonas aeruginosa*	Disruptive effect on biofilm formation, established biofilm	[69]

**Table 4:** Overview of the anti-pathogenic functionalities of COS.

COS Characteristics + Source	[COS]	Strains Used	Observed Effects	References
Chitosan oligosaccharides-from chitosan with DA 89%, DP 3–6 (805)	0.01–0.5%	**Gram-negative:** *Escherichia coli*	Antibacterial activity (anti-growth) and 0.5% completely inhibited the growth of *E. coli*	[126]
Chito-oligosaccharides DA 8.5%, M_w_ 2–30 kDa	0.10%	**Gram-negative:** *Aggregatibacter actinomycetemcomitans* **Gram-positive:** *Streptococcus mutans*	Pathogenic membrane disruption	[102]
Chito-oligosaccharides DA 11% M_w_ <10, <5, <1 kDa	0.1–1%	**Gram-negative:***Escherichia coli*, *Salmonella typhimurium, Pseudomonas aeruginosa* **Gram-positive:***Streptococcus mutans, Micrococcus luteus, Staphylococcus aureus, Staphylococcus epidermidis, Bacillus subtilis*	Higher anti-microbial (anti-growth) effect high-M_w_ COS	[105]
Chitosan M_w_ 5, 8 kDa	0.01–0.5%	**Gram-negative:** *Escherichia coli*	mRNA transcription inhibition	[121]
Chitosans (M_w_ = 1671, 1106, 746, 470, 224, and 28 kDa) Chitosan oligomers (M_w_ = 22, 10, 7, 4, 2, and 1 kDa)	1%	**Gram-negative:** *Escherichia coli, Pseudomonas fluorescens, Salmonella typhimurium, Vibrio parahaemolyticus* **Gram-positive:** *Listeria monocytogenes, Bacillus megaterium, Bacillus cereus, Staphylococcus aureus*	Higher anti-microbial effect (anti-growth) of chitosan compared to COS Chitosan showed stronger bactericidal effects for gram-positive bacteria than gram-negative bacteria	[93]
Chitosan M_w_ < 5 kDa	0.25–1%	**Gram-negative:** *Escherichia coli* **Gram-positive:** *Staphylococcus aureus*	*E. coli* growth inhibition (lower M_w_ more effective) *S*. *aureus* growth inhibition (higher M_w_ more effective)	[94]
Chito-oligosaccharides DA 35.2–37.8% DP 1–6	0.1–0.5%	**Gram-negative:** *Escherichia coli* **Gram-positive:** *Bacillus cereus*	Growth inhibition and cell membrane disruption	[112]
Chito-oligosaccharides DA 3%, DP ~4	0.25–2.5%	**Gram-negative:** *VTEC, EPEC, Desulfovibrio desulfuricans*	Selective anti-adhesion properties	[116]
Chitosan oligosaccharides DA 98.8%, DP 1–16	0.0001–0.5%	**Gram-positive:** *Staphylococcus aureus*	Cell membrane lysis	[113]
Chito-oligosaccharides DA 15–20%, M_W_ <5, <3 kDa	1–5%	**Gram-negative:** *Escherichia coli* **Gram-positive:** *Staphylococcus aureus*	Antimicrobial effect (anti-growth) on *Escherichia coli*, but not Staphylococcus aureus	[95]
Chitosan DA > 90%	0.0004–6.7%	**Gram-negative:***Pseudomonas aeruginosa* (MDR)	Synergy with sulfamethoxazole treatment (anti-growth effect)	[125]
Chitosan oligosaccharides-M_w_= 10,000 Da and 1000 Da)-from chitosan with DA 90–95%	0.5–10 mg/mL	**Gram-negative:** *Vibrio vulnificus*	Higher antimicrobial effect (anti-growth) of water-soluble COS with high molecular weight	[127]
Chitosan DA >90%	0.0004–6.7%	**Gram-possitive:***Staphylococcus aureus* (MDR)	Synergy with several AB treatments (anti-growth effect)	[124]
Chitosans DA 80–85% M_w_ = 628, 591 and 107 kDa Chito-oligosaccharides DA 80–85% M_w_ = <5 and <3 kDa	0.5%	**Gram-negative:** *Escherichia coli, Pseudomonas aeruginosa, Klebsiella pneumoniae* **Gram-positive:** *Staphylococcus aureus, Staphylococcus epidermidis*	Higher antimicrobial effect (anti-growth) of the 3 chitosans	[104]
Chitin (DA 35, M_w_ 388 Da) Chitosan (DA 80, M_w_ 12 Da) COS	0.003–0.1%	**Gram-negative:** *Escherichia coli, Pseudomonas aeruginosa, Salmonella typhimurium, Vibrio cholerae, Shigella dysenteriae, Enterobacter agglomerans, Prevotella melaninogenica, Bacteroides fragilis* **Gram-positive:** *Staphylococcus aureus, Bacillus subtilis, Bacillus cereus*	Higher antimicrobial effect (anti-growth) COS compared to biopolymers	[114]
Chito-oligosaccharides DA ~65%, DP 3–5	2%	**Gram-negative:** *Pseudomonas aeruginosa*	Anti-growth, anti-biofilm functionalities and synergy with azithromycin	[88]
Chito-oligosaccharides DA 9–14%, DP <5–30	1–10%	**Gram-negative:** *Escherichia coli* **Gram-positive:** *Listeria monocytogenes*	High antimicrobial effect (anti-growth) with high DP	[103]
Chito-oligosaccharides-from chitosan with DD 80 and 90%-from chitosan with M_w_ = 5.1, 14.3 and 41.1 kDa	0.002–0.064%	**Gram-negative:** *Escherichia coli, Salmonella typhimurium, Salmonella enteritidis*	High antimicrobial effect (anti-growth) with low DP, potent ferrous chelating activity at low DP	[128]

**Table 5:** Overview of the anti-pathogenic functionalities of GOS.

GOS Characteristics	[GOS]	Strains Used	Observed Effects	References
DP 3–7	0–32 mg/mL	**Gram-negative:** EPEC	Anti-adhesive effect	[138]
DP 3–6	1.56–100 mg/mL	**Gram-negative:** *Vibrio cholerae*	Anti-Ctx	[149]
DP 1–4	2.5 mg/mL	**Gram-negative:** *Salmonella typhimurium*	Anti-adhesive and anti-invasive effect	[139]
DP 2–6	20 mg/mL	**Gram-positive:** *Listeria monocytogenes*	No anti-adhesive and anti-growth effect	[141]
DP 3–6	16 mg/mL	**Gram-negative:** *Cronobacter sakazakii*	Anti-adhesive effect	[142]
-	10–50 mg/mL	**Gram-negative:** *Citrobacter rodentium*	Anti-adhesive effect	[140]

**Table 6:** Overview of the anti-pathogenic functionalities of MOS.

MOS Characteristics	[MOS]	Strains Used	Observed Effects	References
DP 2–6	0.1–0.5 mM	**Gram-negative:** *Escherichia coli*	Anti-adhesive effect	[167]
DP 9	25 μM	**Gram-negative:** *Enterobacter cloacae*	Anti-adhesive effect	[168]
DP 3 MOS	0.13 M–087 M	**Gram-negative:** *Escherichia coli*	Affinity for FimH mannose > MOS	[160]
Partially purified yeast MOS and soluble supernatant fraction of MOS	10–50 mg/mL	**Gram-negative:** *Campylobacter jejuni, Campylobacter coli*	Anti-adhesive effects	[163]
Yeast MOS	6 mg/mL	**Gram-negative:** *Escherichia coli, Salmonella pullorum*	Anti-adhesive effect (less effective than yeast cell walls)	[166]

**Table 7:** Overview of the anti-pathogenic functionalities of POS.

POS Characteristics + Source	[POS]	Strains Used	Observed Effects	References
M_w_ 1–4 kDa Citrus (high methylation) Apple (low methylation)	10 mg/mL	**Gram-negative:***Escherichia coli* O157:H7 Shiga toxin	Inhibition of host cell infiltration of Stx	[185]
M_w_ 1–12 kDa Panax ginseng	0.01–0.5 mg/mL	**Gram-negative:** *Aggregatibacter actinomycetemcomitans* **Gram-positive:** *Staphylococcus aureus*	Anti-adhesive effect	[189]
DP 2–3 Orange peel	2.5 mg/mL	**Gram-negative:** EPEC, VTEC, *Desulfovibrio desulfuricans*	Anti-adhesive effect	[186]
GalA:Rhamnose 1:1 Albedo of orange peel	0.05–2.5 mg/mL	**Gram-negative:** *Campylobacter jejuni*	Inhibited Caco-2 cell invasion	[177]
0.2–6 kDa 93.6% Uronic acid Haw	1–10 mg/mL	**Gram-negative:** *Escherichia coli*	Antimicrobial activity dependent on concentration and low pH	[178]
DP 6–19 Orange peel	1–100 mg/mL	**Gram-negative:***Escherichia coli* **Gram-positive:***Staphylococcus aureus*, *Bacillus subtilis*	Antimicrobial activity-low Mw more effective	[176]
Apple, citrus, polygalacturonic acid	0.1 mg/mL	**Gram-negative:** *Escherichia coli* **Gram-positive:** *Staphylococcus aureus*	Growth inhibition, potentially through CO_2_ radical production	[184]
M_w_ 9–73 kDa Orange peel	0.005–5 mg/mL	**Gram-negative:** Shigatoxigenic *Escherichia coli*	Anti-adhesive effect, direct interaction with Stx	[188]

## Abbreviations

2-FL	2-Fucosyllactose
3-FL	3-Fucosyllactose
3-SL	3-Sialyllactose
6-SL	6-Sialyllactose
α1,2-fucose	FUT2
α1,4-fucose	FUT3
AHL	Acyl homoserine lactones
AOS	Alginate oligosaccharide
AP	Acetylation pattern
COS	Chito-oligosaccharides/chitosan oligosaccharides
Ctx	*Vibrio cholerae* toxin
DA	Degree of acetylation
DD	Degree of deacetylation
DP	Degree of polymerization
EPEC	Enteropathogenic *Escherichia coli*
EPS	Extracellular polymeric substances
FO	Fucosylated oligosaccharides
FOS	Fructo-oligosaccharides
Gal	Galactose
GBS	*Group B Streptococcus*
Gb3	Globotriaosylceramide
GC	Guluronic content
Glc	Glucose
GlcN	β-1,4-linked d-glucosamine
GlcNAc	N-acetylglucosamine
GOS	Galacto-oligosaccharides
HMOs	Human milk oligosaccharides
HMWF	High-molecular weight fraction
LMWF	Low-molecular weight fraction
LNFP I	Lacto-N-fucopentaose I
LNT	Lacto-N-tetraose
LSTa	LS-tetrasaccharide a
MOS	Mannan-oligosaccharides
NDOs	Non-digestible oligosaccharides
POS	Pectic oligosaccharides
QS	Quorum-Sensing
Stx	Shiga toxin
UPEC	UroPathogenic *Escherichia coli*
VTEC	Verocytotoxin-producing *Escherichia coli*
